# C-terminal and intact FGF23 in kidney transplant recipients and their associations with overall graft survival

**DOI:** 10.1186/s12882-021-02329-7

**Published:** 2021-04-08

**Authors:** Chang Chu, Saban Elitok, Shufei Zeng, Yingquan Xiong, Carl-Friedrich Hocher, Ahmed A. Hasan, Bernhard K. Krämer, Berthold Hocher

**Affiliations:** 1 Fifth Department of Medicine (Nephrology/ Endocrinology/ Rheumatology), University Medical Centre Mannheim, University of Heidelberg, Heidelberg, Germany; 2 Department of Nephrology, Charité - Universitätsmedizin Berlin, Campus Mitte, Berlin, Germany; 3 Clinic for Nephrology and Endocrinology, Klinikum Ernst von Bergmann, Potsdam, Germany; 4 Institute of Pharmacy, Free University of Berlin, Berlin, Germany; 5 Department of Biochemistry, Faculty of Pharmacy, Zagazig University, Zagazig, Egypt; 6 European Center for Angioscience, Medical Faculty Mannheim of the University of Heidelberg, Heidelberg, Germany; 7 Mannheim Institute for Innate Immunoscience, Medical Faculty Mannheim of the University of Heidelberg, Mannheim, Germany; 8 Key Laboratory of Study and Discovery of Small Targeted Molecules of Hunan Province, School of Medicine, Hunan Normal University, Changsha, China; 9 Reproductive and Genetic Hospital of CITIC-Xiangya, Changsha, China; 10 IMD Institut für Medizinische Diagnostik Berlin-Potsdam GbR, Berlin, Germany

**Keywords:** Fibroblast growth factor 23, Graft loss, All-cause mortality, Kidney transplant recipient

## Abstract

**Background:**

Increased fibroblast growth factor 23 (FGF23) is a risk factor for mortality, cardiovascular disease, and progression of chronic kidney disease. Limited data exist comparing the association of either c-terminal FGF23 (cFGF23) or intact FGF23 (iFGF23) in kidney transplant recipients (KTRs) with overall (all-cause) graft loss.

**Methods:**

We conducted a prospective observational cohort study in 562 stable kidney transplant recipients. Patients were followed for graft loss and all-cause mortality for a median follow-up of 48 months.

**Results:**

During a median follow-up of 48 months, 94 patients had overall graft loss (primary graft loss or death with functioning graft). Both cFGF23 and iFGF23 concentrations were significantly higher in patients with overall graft loss than those without (24.59 [11.43–87.82] versus 10.67 [5.99–22.73] pg/ml; *p* < 0.0001 and 45.24 [18.63–159.00] versus 29.04 [15.23–60.65] pg/ml; *p* = 0.002 for cFGF23 and iFGF23, respectively). Time-dependent ROC analysis showed that cFGF23 concentrations had a better discriminatory ability than iFGF23 concentrations in predicting overall (all-cause) graft loss. Cox regression analyses adjusted for risk factors showed that cFGF23 (HR for one unit increase of log transformed cFGF23: 1.35; 95% CI, 1.01–1.79; *p* = 0.043) but not iFGF23 (HR for one unit increase of log transformed iFGF23: 0.97; 95% CI, 0.75–1.25; *p* = 0.794) was associated with the overall graft loss.

**Conclusion:**

Elevated cFGF23 concentrations at baseline are independently associated with an increased risk of overall graft loss. iFGF23 measurements were not independently associated with overall graft loss. The cFGF23 ELISA might detect bioactive FGF23 fragments that are not detected by the iFGF23 ELISA.

**Supplementary Information:**

The online version contains supplementary material available at 10.1186/s12882-021-02329-7.

## Background

Kidney transplantation is the preferred replacement therapy for end-stage kidney disease (ESKD) [1–3]. The burden of cardiovascular disease (CVD) in ESKD is reduced after kidney transplantation, however, it remains one of the leading causes of premature mortality and allograft loss in kidney transplant recipients (KTRs) [4]. More than half deaths among KTRs are directly attributable to cardiovascular disease [5, 6].

Fibroblast growth factor 23 (FGF23) is an osteocyte-derived hormone and is involved in mineral-bone homeostasis by regulating serum phosphate, parathyroid hormone (PTH), and 1,25-(OH)_2_- VD3 [7–9]. The kidney is the principal target for FGF23, and the major function of this hormone is to regulate phosphate reabsorption and synthesis of 1,25(OH)_2_D [9]. FGF23 is an approximately 32-kD (251 amino acids) protein with a N-terminal region that contains the FGF homology domain and a novel 71-amino acid C-terminus [10]. Two types of assays for determination of FGF23 concentrations in human are currently available. Intact FGF23 (iFGF23) assay binds two epitopes that flank the proteolytic cleavage site that lays between amino acids 179 and 180, hence presumably detecting only biologically active, full-length FGF23 (∼32 kDa) [11], whereas the C-terminal FGF23 (cFGF23) assay binds to epitopes within the C-terminal region of the FGF23 protein and therefore detects both full-length and processed C-terminal fragment (∼14 kDa) [12].

Prior clinical studies revealed a close relationship between FGF23 and the cardiovascular morbidity and mortality in both the CKD populations and the general population [13, 14]. In KTRs, circulating FGF23 concentrations seems to be an independent biomarker for cardiovascular and all-cause mortality and allograft loss [15, 16]. However, most studies reported associations of FGF23 with clinical outcomes have used cFGF23 assays [15–19], although it was thought that the iFGF23 assays should be superior, because they just detect the full-length FGF23 molecule and not a mixture of full-length FGF23 and degradation products [20].

Limited data exist with regard to head-to-head comparisons cFGF23 and iFGF23 measurements in kidney transplant recipients with respect to graft loss and all-cause mortality. Thus, we carried out this study to compare the predictive power of both iFGF23 and cFGF23 with composite outcomes of graft loss and all-cause mortality in KTRs.

## Materials and methods

### Study participants and design

This prospective observational cohort study comprised prevalent kidney transplant recipients who had kidney transplantation before 15th October 2012 and came for routine check-ups at the transplant outpatient clinic Charité-Mitte, Berlin, Germany.

Patients with active infections, malignancy, acute rejection, a recent cardiovascular event at the time of study inclusion or those unwilling to participate were excluded. Patients were prospectively followed up for a median of 4.0 years (IQR 3.93–4.04). Prespecified endpoint was defined as overall renal graft loss included both functional grafts lost due to recipient death and death-censored graft loss. Graft loss was defined as return to dialysis, graft removal, re-transplantation, based on the judgment of the treating physicians. The primary outcome was defined as overall (all-cause) graft loss comprising primary renal allograft loss and death with functioning renal allograft. The study protocol was approved by the ethics committee of the Charité - Universitätsmedizin Berlin (Charitéplatz 1, 10,117 Berlin; Germany) and informed consent was obtained from all participants. All methods were carried out in accordance with relevant guidelines and regulations.

### Data source and laboratory measurements

Baseline characteristics of KTRs and kidney donors, such as age, gender, recipients’ primary kidney diseases and post-transplant duration, cold ischemia time, human leukocyte antigen mismatches, panel reactive antibodies (PRA) were extracted from hospital records.

Blood and urine samples were collected from patients during routine visits from April 2012 until December 2012. Baseline laboratory measurements, such as creatinine, serum albumin, total cholesterol, fasting blood glucose, calcium, phosphorus, as well as urinary protein excretion were measured in the central clinical laboratory of the Charité Universitätsmedizin Berlin, Germany. Baseline estimated glomerular filtration rate (eGFR) was calculated using the CKD-Epidemiology Collaboration equation.

### cFGF23 and iFGF23 measurements

cFGF23 and iFGF23 were measured with commercially available ELISA [FGF23 (C-terminal) multi-matrix ELISA, cat. no. BI-20702, Biomedica, Austria and FGF23 (intact) human ELISA, cat. no.BI-20700, Biomedica, Austria) according to the instructions of the manufacturer. The average intra-and inter-assay coefficients of variation were ≤ 12% and ≤ 10% for cFGF23 assay (described in detail on https://www.bmgrp.com/wp-content/uploads/2019/03/bi-20702-fgf23-elisa-validation-data-150306.pdf), and ≤ 8 and ≤ 6% for iFGF23 assay (described in detail on https://www.bmgrp.com/wp-content/uploads/2019/06/BI-20700-FGF23-Intact-ELISA-Validation-Data-CE-190808.pdf). All samples were measured in duplicate and all assays were subject to regular quality control.

### Statistical analysis

Descriptive statistics of baseline characteristics are presented as means (standard deviation) for normally distributed data or as medians (interquartile ranges) for not normally distributed variables. Categorical data are presented as numbers (%). Statistical differences between baseline characteristics were analyzed using a Student’s t test or Mann Whitney U test, or χ^2^ test as appropriate. Graft survival and time-to-event analysis was estimated by the Kaplan-Meier, differences were evaluated with a stratified log-rank test. Univariate analysis was used to determine the association between the FGF23 concentration and graft survival. Multivariable-adjusted Cox regression analysis was used to estimate the simultaneous effects of established and emerging risk factors on graft loss and mortality. Model 1 was adjusted for eGFR, model 2 was adjusted for eGFR, gender, age; model 3 was adjusted for eGFR, gender, age, time post-transplantation, hemoglobin, albumin, donor’s age, cold ischemia time, log serum calcium, log serum phosphorus, log parathyroid hormone, urinary protein excretion, model 4 was adjusted for the same factors as model 3, plus inflammation marker (C-reactive protein), model 5 was adjusted for the same factors as model 3, plus iron status markers (mean corpuscular volume and ferritin), model 6 was fully adjusted model, including all adjustments. The Bland-Altman plots were used to evaluate the agreement of mean difference between cFGF23 and iFGF23. *P* value < 0.05 was regarded statistically significant. Statistical analyses other than time-dependent ROC were performed using SPSS version 25.0 (Chicago, IL, USA). Time-dependent ROC curves was conducted with R software (version 4.0.4, 2021-02-14), in addition, *timeROC* (version 0.4) and *ggplot2* (version 3.3.3) packages were included.

### Meta-analysis

Details of the data sources, search strategy, study selection, eligibility criteria, data extraction, quality assessment and analysis method were described in supplementary file.

## Results

### Patient characteristics

Demographic and clinical data of the cohort are shown in the Table 1. The mean time post-transplantation’ until study entry (baseline evaluation) was 5.89 years. No patients were lost to follow-up. Overall, recipients without overall graft loss were younger, had younger donors and less cold ischemia time, also showed better status of serum and urinary parameters, such as higher hemoglobin and albumin, lower creatinine and parathyroid hormone, etc. Baseline median cFGF23 (*n* = 546) and iFGF23 (*n* = 502) were 12.57 pg/ml (interquartile range, 6.38–27.38 pg/ml) and 30.94 pg/ml (interquartile range, 15.43–68.98 pg/ml), respectively. Both median intact and c-terminal FGF23 levels were significant lower in the survival group compare to the patients who had graft loss or died (*p* = 0.002).

**Table 1:** Baseline characteristics of the cohort

Variables	All patients (*n* = 562)	Death/graft loss (*n* = 94)	No event (*n* = 468)	*P*
Age at study entry (years)	54.6 (44.5–66.9)	62.5 (54.0–71.6)	53.4 (43.0–64.2)	< 0.0001
Male, n (%)	345 (61.4%)	68 (72.3%)	227 (59.2%)	0.017
Primary kidney diseases, n (%)				0.888
Glomerulonephritis	228 (40.6%)	38 (40.4%)	190 (40.6%)	
Tubulointerstitial disease	54 (9.6)	9 (9.6%)	45 (9.6%)	
Polycystic renal disease	70 (12.5%)	10 (10.6%)	60 (12.8%)	
Dysplasia and hypoplasia	8 (1.4%)	1 (1.1%)	7 (1.5%)	
Diabetic nephropathy	18 (3.2%)	3 (3.2%)	15 (3.2%)	
Hypertensive nephropathy	16 (2.8%)	2 (2.1%)	14 (3.0%)	
Other or unknown cause	168 (29.9)	31 (33.0%)	137 (29.3%)	
Time post-transplantation (years)	5.89 (2.90–10.48)	6.93 (3.65–12.23)	5.69 (2.88–10.37)	0.063
Time on dialysis (month)	46.0 (19.0–75.0)	50.0 (21.5–73.5)	44.0 (19.0–76.0)	0.503
Donor age (years)	51.0 (40.0–61.0)	54.0 (42.0–68.0)	50.0 (39.0–59.0)	0.013
Cold ischemia time (hours)	8.51 (3.24–14.04)	9.92 (6.42–18.16)	8.18 (3.02–13.73)	0.023
Hemoglobin (g/dl)	12.71 ± 1.76	12.20 ± 1.78	12.81 ± 1.74	0.002
Serum albumin (g/dl)	4.55 ± 0.35	4.46 ± 0.36	4.57 ± 0.35	0.04
Total cholesterol (mg/dl)	217.0 (186.0–254.0)	210.0 (171.5–247.3)	218.0 (187.0–254.8)	0.221
Serum creatinine (mg/dl)	1.57 (1.26–2.06)	2.23 (1.42–3.02)	1.52 (1.24–1.94)	< 0.0001
eGFR (mL/min/1.73 m^2^)	43.0 (31.0–58.0)	30.0 (21.0–50.5)	44.5 (34.0–58.0)	< 0.0001
Serum calcium (mmol/L)	2.47 (2.37–2.59)	2.42 (2.33–2.57)	2.48 (2.39–2.59)	0.014
Serum phosphorus (mmol/L)	0.86 (0.71–1.02)	0.99 (0.77–1.23)	0.84 (0.69–0.98)	< 0.0001
Parathyroid hormone (pg/ml)	79.18 (49.91–133.70)	104.9 (58.76–206.3)	76.34 (49.06–126.60)	0.002
Intact FGF23 (pg/ml)	30.94 (15.43–68.98)	45.24 (18.63–159.0)	29.04 (15.23–60.65)	0.002
C terminal FGF23 (pg/ml)	12.57 (6.38–27.38)	24.59 (11.43–87.82)	10.67 (5.99–22.73)	< 0.0001
Urinary protein excretion (mg/24 h)	163.0 (99.0–306.5)	265.5 (129.5–691.5)	153.0 (96.0–274.0)	0.0001

Data are given as median (interquartile ranges) or number (%). Baseline characteristics of the study population. Patients were followed for graft loss and all-cause mortality for a follow-up of 48 months. The composite end-point (event) was defined as graft loss or death due to any reason

*eGFR* estimated glomerular filtration rate

### cFGF23 and clinical outcomes

**Fig. 1:**
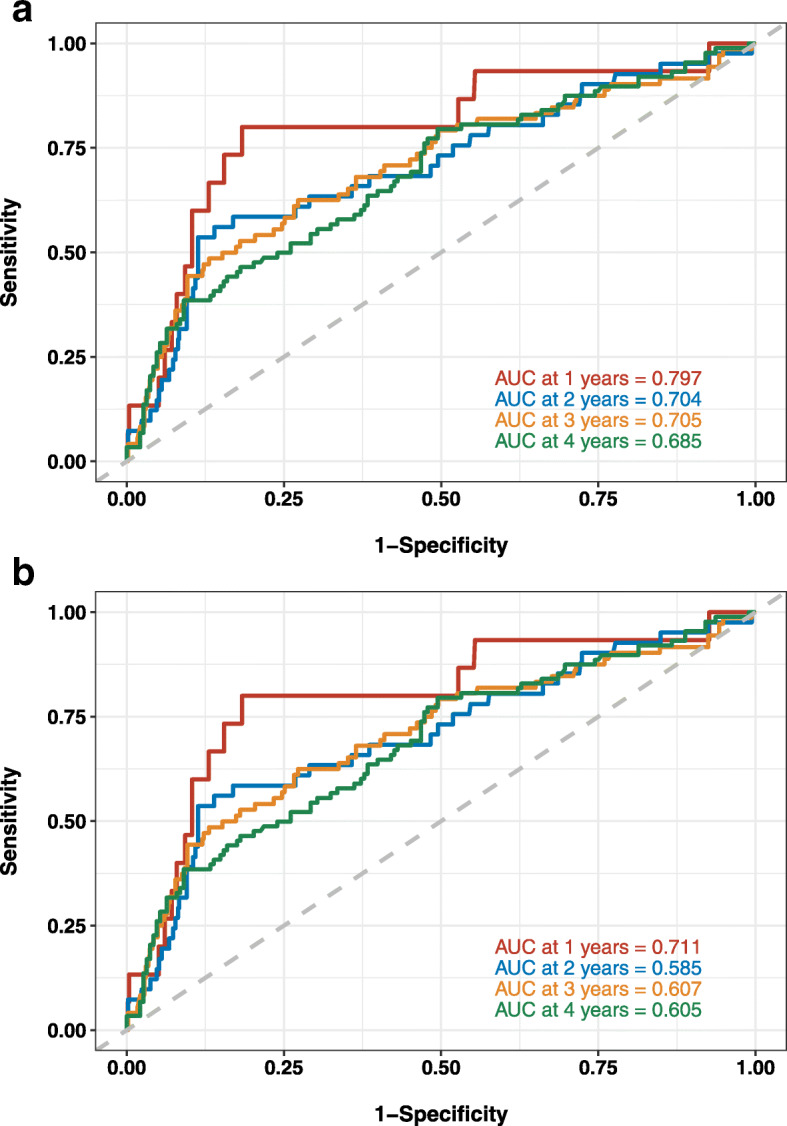
**a** Time-dependent Receiver operating characteristic (ROC) curves of cFGF23 for composite outcome of all-cause mortality and graft loss. **b** Time-dependent Receiver operating characteristic (ROC) curves of iFGF23 for composite outcome of all-cause mortality and graft loss. AUC, area under the curve

During a median follow-up of 4.0 years (interquartile range, 3.93 to 4.04 years), 94 patients suffered from overall graft loss. Time-dependent ROC analysis showed that cFGF23 concentrations had a better discriminatory ability than iFGF23 concentrations in predicting overall (all-cause) graft loss. Plots of AUROCs (area under the ROC) at 1-year, 2-year, 3-year, 4-year of cFGF23 and iFGF23 for overall graft loss are shown in Fig. 1. The AUROCs of cFGF23 vs. iFGF23 at 1-year, 2-year, 3-year, 4-year were 0.797 vs. 0.711, 0.704 vs. 0.585, 0.705 vs. 0.607 and 0.685 vs. 0.605, respectively.

**Fig. 2:**
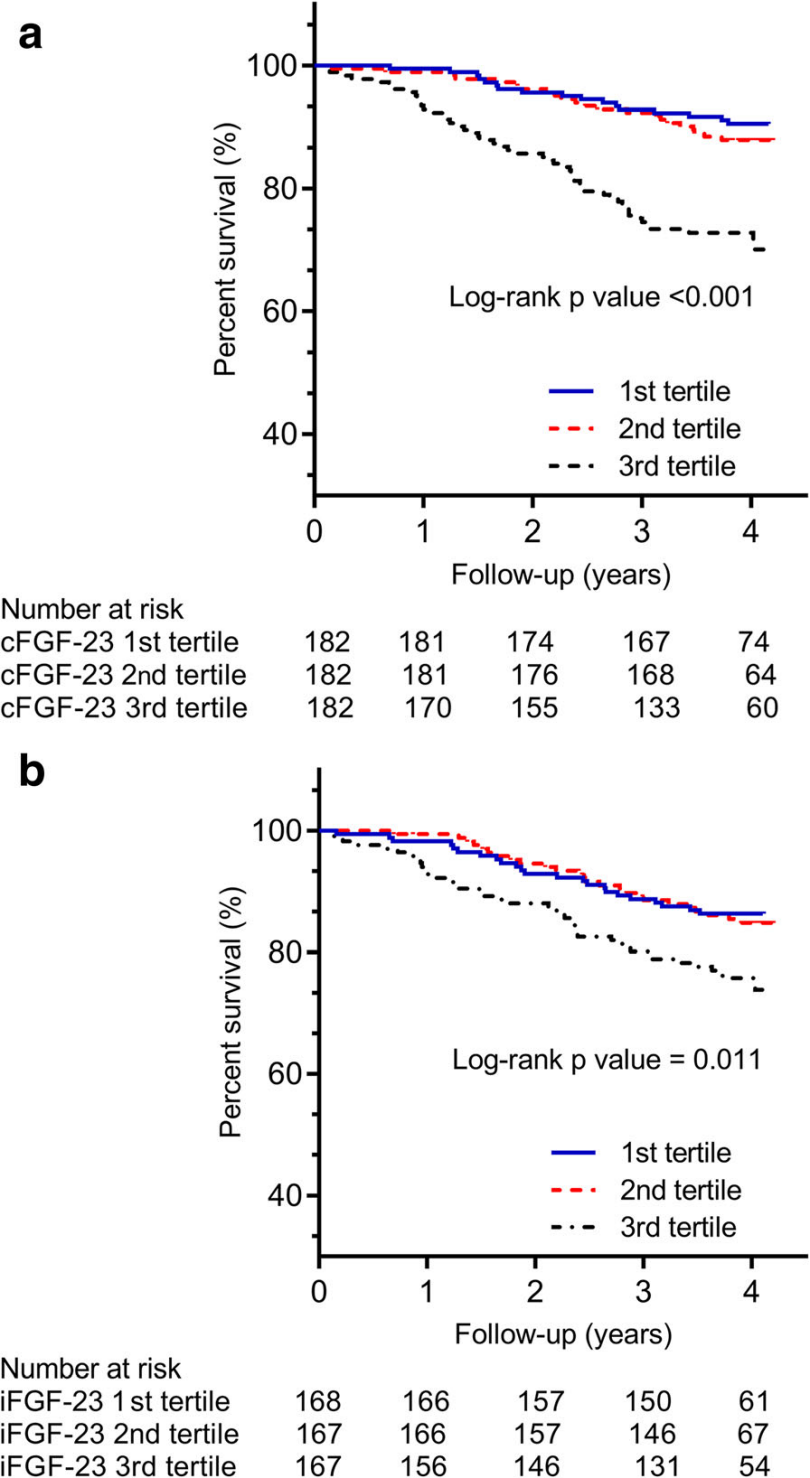
Kaplan-Meier curves for composite outcome of all-cause mortality and graft loss. Patients were subdivided into 3 subgroups based on the tertile of FGF23; **a** cFGF23. **b** iFGF23

According to cFGF23 concentrations, patients were divided into tertiles. Kaplan-Meier survival plots demonstrated a significant lower risk of composite endpoint of patients in the 3rd tertile of cFGF23 (log-rank test, *P* < 0.001) (Fig. 2a).

In Cox regression models, increasing log transformed cFGF23 was independently associated with the composite endpoint in the univariate Cox regression analysis. We aimed to determine whether cFGF23 was independently associated with overall (all-cause) graft loss and explored possible variations in this association. We used Cox regression models with a stepwise selection of variables that associated with death or graft failure. cFGF23 remained a significant predictor of overall (all-cause) graft loss, even after full adjustments (Table 2). However, when analyzing the individual components of the composite endpoint separately, the association of cFGF23 with the either all-cause mortality or graft loss after full adjustments was not significant (supplementary Table 4). The effects of the individual components of the composite endpoint went into the same direction as the composite endpoint, the effect of graft loss on the composite endpoint seems to be more pronounced.

**Table 2:** Cox regression analysis for composite outcome of all-cause mortality and graft loss

Variable	Composite outcome	
	HR (95% CI)	P
***Log C-terminal FGF23***		
Univariate analysis	1.46 (1.31–1.63)	< 0.001
Model 1	1.23 (1.07–1.42)	0.005
Model 2	1.28 (1.09–1.50)	0.003
Model 3	1.35 (1.03–1.76)	0.029
Model 4	1.37 (1.03, 1.81)	0.030
Model 5	1.33 (1.01, 1.75)	0.040
Model 6	1.35 (1.01, 1.79)	0.043
***Log intact FGF23***		
Univariate analysis	1.28 (1.14–1.44)	< 0.001
Model 1	1.05 (0.92–1.21)	0.473
Model 2	1.06 (0.92–1.23)	0.425
Model 3	1.03 (0.81–1.31)	0.833
Model 4	0.99 (0.77, 1.28)	0.936
Model 5	0.99 (0.77, 1.26)	0.920
Model 6	0.97 (0.75, 1.25)	0.794

Model 1: eGFR; Model 2: eGFR, gender, age; Model 3: eGFR, gender, age, time post-transplantation, hemoglobin, albumin, donor’s age, cold ischemia time, log serum calcium, log serum phosphorus, log parathyroid hormone, urinary protein excretion; Model 4: model 3 + CRP; Model 5: model 3 + MCV + ferritin; Model 6: model 3 + CRP + MCV + ferritin

*HR* hazard ratio, *95%CI* 95% confidence interval, *eGFR* estimated glomerular filtration rate, *CRP* C-reactive protein, *MCV* mean corpuscular volume

### iFGF23 and clinical outcomes

Same analyses as cFGF23 were conducted in iFGF23. Plots of AUROCs (area under the ROC) at 1-year, 2-year, 3-year, 4-year of cFGF23 and iFGF23 for overall graft loss are shown in Fig. 1.

Kaplan-Meier survival analysis indicated that patients with the 3rd tertile iFGF23 concentrations had a significantly lower risk of composite endpoint (log-rank test, *P* = 0.011) (Fig. 2b).

In unadjusted Cox regression analysis, log transformed iFGF23 showed significant association with the composite outcome (HR, 1.28; 95% CI, 1.14–1.44; *P* < 0.001). However, when adjusted for eGFR, the association was no longer statistically significant (*P* = 0.473). log transformed iFGF23 did not consistently show a significant association with composite endpoint after additional adjustments were added (Table 2).

### Comparison of cFGF23 and iFGF23

**Fig. 3:**
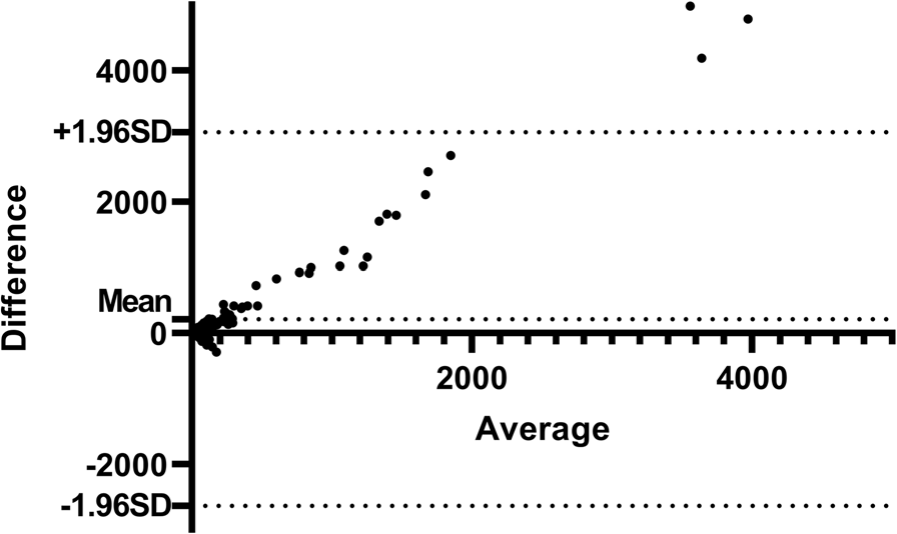
Bland-Altman plot of cFGF23 and iFGF23

Bland-Altman plots revealed a significant difference between cFGF23 and iFGF23 (Fig. 3). In our cohort, the Bland-Altman analysis revealed systematic differences between iFGF23 and cFGF23 Elisa’s, indicating that both assays indeed measure different forms of FGF23.

## Discussion

This prospective cohort study is the first study in KTRs simultaneously analyzing baseline iFGF23 and cFGF23. Our study demonstrated - after adjusting for risk factors – that only cFGF23 was independently associated with the overall graft loss, and this was consistent with the results of a meta-analysis analyzing all so far reported cFGF23 studies [15–19] and our data in KTRs. Furthermore, we found that FGF23 correlated negatively with eGFR in this cohort, moreover, cFGF23 concentration showed a stronger correlation with eGFR than iFGF23 (r_s_ = − 0.475, *p* < 0.001 and r_s_ = − 0.385, p < 0.001 for cFGF23 and iFGF23, respectively.), which indicate that cFGF23 may capture risk of reduced kidney function, which is not similarly captured by iFGF23.

Only one published study investigated the association between iFGF23 and the overall graft loss in KTRs so far [21]. In line with our study, the positive association of iFGF23 with poor outcome in the unadjusted analysis was no longer significant after adjusted for risk factors in the multivariate analysis (*P* = 0.97), however, this study did not analyze cFGF23.

FGF23 is a 251 amino acid peptide with a 24 amino acid signal peptide and 227 residues forming the mature FGF-23 structure [22]. FGF23 is likely to be cleaved by subtilisin-like proprotein – recognizing amino acids 179Arg and 180Ser as cleavage motive [23, 24]. After cleavage, biologically inactive peptides are produced (an inactive N-terminal (amino acids 25–179) and an inactive C-terminal fragment (amino acids 180–251). More yet unknown clearage motives may exist. Nowadays, the commercial iFGF23 assays recognize two epitopes that flank the proteolytic cleavage site that lays between amino acids 179 and 180, thus ensuring that only biologically active, full-length FGF23 is detected, whereas the cFGF23 assays recognize the biologically active full-length intact FGF23 (iFGF23) hormone as well as its C-terminal fragments [11] (Supplementary Figure 1).

Under physiological conditions, iFGF23 is the most abundant form of circulating FGF23 peptides [25, 26]. We initially hypothesized that the biologically active FGF23 is better associated with clinical outcomes and might reflect better the biological properties of FGF23. However, the opposite finding seems to be the case. A very recent study suggests that the iron status and inflammation may have confounded the associations of iFGF23 with clinical outcomes [27]. Under healthy conditions increased transcription due to iron deficiency and/or inflammation are matched with increased cleavage to maintain normal circulating levels of full-length FGF23, resulting in elevated concentrations of circulating cFGF23, but normal concentrations of iFGF23 and hence a better correlation of cFGF23 with outcomes as compared with iFGF23 measurements.

This study [27] stimulated us to analyze whether the iron status may also influence our findings in KTRs. We thus conducted a series of multivariate models. After adjusting for kidney function (model 1), the association between iFGF23 and the overall graft loss was no longer significant. In the subsequent analysis, more covariates were added to the model, model 4 considered C-reactive protein (CRP), which represented inflammation on top of conventional risk factors, model 5 added mean corpuscular volume (MCV) and ferritin as markers of the iron status. Model 6 was fully adjusted for all risk factors (Table 2). cFGF23, but not iFGF23, showed consistently significant association with the composite outcomes. Thus, our data rather suggest that fragments of FGF23 are more important than the full length FGF23 molecule for predicting outcome in stable kidney transplant recipients. Alternatively, yet not identified confounding factors in humans who were just described in animal models [28, 29] might likewise modify the association as it was the case for iron deficiency [11].

Our study consists of a well-defined cohort of KTRs without loss of follow-up. We determined both serum iFGF23 and cFGF23 levels and performed three independent statistical methods (Time-dependent ROC curve analysis, Kaplan-Meier analysis and Cox proportional-hazards model) to assess the association between FGF23 and overall graft loss. We acknowledge several limitations of this study. First, iFGF23 as well as cFGF23 concentrations were only measured once at study entry, hence it is impossible to investigate the alteration over time in this cohort. Second, concerning the limited number of endpoint events, we did not conduct subgroup analyses of graft loss and death separately. Third, the binding sites of the antibodies in the used cFGF23 ELISAs were not identical, because they came from different companies (Immutopics versus Biomedica). Although data coming for studies using either the cFGF23 Immutopics Elisa or the cFGF23 Elisa from Biomedica yielded qualitatively identical results, the differences in the binding sites might have influenced the ability of the individual ELISA to detect breakdown-products of FGF23 (see supplementary Figure 1). Furthermore, this cross-sectional study can only show the association between baseline cFGF23 and endpoints, further studies analyzing just potential degradation products of full length FGF23 are needed.

## Conclusion

Consistent with prior studies, we also demonstrated that baseline serum cFGF23 was independently associated with the overall graft loss in KTRs. Simultaneously measured iFGF23, however, was not associated with the composite outcome after adjusting for risk factors. The cFGF23 ELISA might detect bioactive FGF23 fragments that are not detected by the iFGF23 ELISA.

## Supplementary Information

**Additional file 1.**

## Data Availability

The datasets used and/or analyzed during the current study are available from the corresponding author on reasonable request.

## References

[1] Schnuelle P, Lorenz D, Trede M, Van Der Woude FJ (1998). Impact of renal cadaveric transplantation on survival in end-stage renal failure: evidence for reduced mortality risk compared with hemodialysis during long-term follow-up. J Am Soc Nephrol.

[2] Port FK, Wolfe RA, Mauger EA, Berling DP, Jiang K (1993). Comparison of survival probabilities for dialysis patients vs cadaveric renal transplant recipients. JAMA..

[3] Ojo AO, Port FK, Wolfe RA, Mauger EA, Williams L, Berling DP (1994). Comparative mortality risks of chronic dialysis and cadaveric transplantation in black end-stage renal disease patients. Am J Kidney Dis.

[4] Rangaswami J, Mathew RO, Parasuraman R, Tantisattamo E, Lubetzky M, Rao S, Yaqub MS, Birdwell KA, Bennett W, Dalal P, Kapoor R, Lerma EV, Lerman M, McCormick N, Bangalore S, McCullough PA, Dadhania DM (2019). Cardiovascular disease in the kidney transplant recipient: epidemiology, diagnosis and management strategies. Nephrol Dial Transplant.

[5] Yeo FE, Villines TC, Bucci JR, Taylor AJ, Abbott KC (2004). Cardiovascular risk in stage 4 and 5 nephropathy. Adv Chronic Kidney Dis.

[6] Ojo AO (2006). Cardiovascular complications after renal transplantation and their prevention. Transplantation..

[7] Krajisnik T, Bjorklund P, Marsell R, Ljunggren O, Akerstrom G, Jonsson KB (2007). Fibroblast growth factor-23 regulates parathyroid hormone and 1alpha-hydroxylase expression in cultured bovine parathyroid cells. J Endocrinol.

[8] Kuro OM, Moe OW (2017). FGF23-alphaKlotho as a paradigm for a kidney-bone network. Bone..

[9] Shimada T, Kakitani M, Yamazaki Y, Hasegawa H, Takeuchi Y, Fujita T, Fukumoto S, Tomizuka K, Yamashita T (2004). Targeted ablation of Fgf23 demonstrates an essential physiological role of FGF23 in phosphate and vitamin D metabolism. J Clin Invest.

[10] Liu S, Quarles LD (2007). How fibroblast growth factor 23 works. J Am Soc Nephrol.

[11] Wolf M, White KE (2014). Coupling fibroblast growth factor 23 production and cleavage: iron deficiency, rickets, and kidney disease. Curr Opin Nephrol Hypertens.

[12] Jonsson KB, Zahradnik R, Larsson T, White KE, Sugimoto T, Imanishi Y, Yamamoto T, Hampson G, Koshiyama H, Ljunggren Ö, Oba K, Yang IM, Miyauchi A, Econs MJ, Lavigne J, Jüppner H (2003). Fibroblast growth factor 23 in oncogenic osteomalacia and X-linked hypophosphatemia. N Engl J Med.

[13] Sarmento-Dias M, Santos-Araujo C, Poinhos R, Oliveira B, Silva IS, Silva LS (2016). Fibroblast growth factor 23 is associated with left ventricular hypertrophy, not with uremic vasculopathy in peritoneal dialysis patients. Clin Nephrol.

[14] Mirza MA, Larsson A, Melhus H, Lind L, Larsson TE (2009). Serum intact FGF23 associate with left ventricular mass, hypertrophy and geometry in an elderly population. Atherosclerosis..

[15] Baia LC, Humalda JK, Vervloet MG, Navis G, Bakker SJ, de Borst MH, NIGRAM Consortium (2013). Fibroblast growth factor 23 and cardiovascular mortality after kidney transplantation. Clin J Am Soc Nephrol.

[16] Wolf M, Molnar MZ, Amaral AP, Czira ME, Rudas A, Ujszaszi A, Kiss I, Rosivall L, Kosa J, Lakatos P, Kovesdy CP, Mucsi I (2011). Elevated fibroblast growth factor 23 is a risk factor for kidney transplant loss and mortality. J Am Soc Nephrol.

[17] Bienaime F, Dechartres A, Anglicheau D, Sabbah L, Montgermont P, Friedlander G (2017). The association between fibroblast growth factor 23 and renal transplantation outcome is modified by follow-up duration and glomerular filtration rate assessment method. Kidney Int Rep.

[18] Eisenga MF, van Londen M, Leaf DE, Nolte IM, Navis G, Bakker SJL, de Borst MH, Gaillard CAJM (2017). C-terminal fibroblast growth factor 23, Iron deficiency, and mortality in renal transplant recipients. J Am Soc Nephrol.

[19] Prakobsuk S, Sirilak S, Vipattawat K, Taweesedt PT, Sumethkul V, Kantachuvesiri S, Disthabanchong S (2017). Hyperparathyroidism and increased fractional excretion of phosphate predict allograft loss in long-term kidney transplant recipients. Clin Exp Nephrol.

[20] Bozentowicz-Wikarek M, Owczarek A, Kocelak P, Olszanecka-Glinianowicz M, Wiecek A, Chudek J (2016). C-terminal to intact fibroblast growth factor 23 ratio in relation to estimated glomerular filtration rate in elderly population. Kidney Blood Press Res.

[21] Doi Y, Hamano T, Ichimaru N, Tomida K, Obi Y, Fujii N, Yamaguchi S, Oka T, Sakaguchi Y, Matsui I, Kaimori JY, Abe T, Imamura R, Takahara S, Tsubakihara Y, Nonomura N, Isaka Y (2020). Serum phosphate levels modify the impact of parathyroid hormone levels on renal outcomes in kidney transplant recipients. Sci Rep.

[22] Kocelak P, Olszanecka-Glinianowicz M, Chudek J (2012). Fibroblast growth factor 23--structure, function and role in kidney diseases. Adv Clin Exp Med.

[23] Shimada T, Hasegawa H, Yamazaki Y, Muto T, Hino R, Takeuchi Y, Fujita T, Nakahara K, Fukumoto S, Yamashita T (2004). FGF-23 is a potent regulator of vitamin D metabolism and phosphate homeostasis. J Bone Miner Res.

[24] Benet-Pages A, Lorenz-Depiereux B, Zischka H, White KE, Econs MJ, Strom TM (2004). FGF23 is processed by proprotein convertases but not by PHEX. Bone..

[25] Yamazaki Y, Okazaki R, Shibata M, Hasegawa Y, Satoh K, Tajima T, Takeuchi Y, Fujita T, Nakahara K, Yamashita T, Fukumoto S (2002). Increased circulatory level of biologically active full-length FGF-23 in patients with hypophosphatemic rickets/osteomalacia. J Clin Endocrinol Metab.

[26] Larsson T, Nisbeth U, Ljunggren O, Juppner H, Jonsson KB (2003). Circulating concentration of FGF-23 increases as renal function declines in patients with chronic kidney disease, but does not change in response to variation in phosphate intake in healthy volunteers. Kidney Int.

[27] Sharma S, Katz R, Bullen AL, Chaves PHM, de Leeuw PW, Kroon AA, et al. Intact and C-terminal FGF23 assays-do kidney function, inflammation, and low iron influence relationships with outcomes? J Clin Endocrinol Metab. 2020;105(12). 10.1210/clinem/dgaa665 PMC7571450 32951052

[28] Lang F, Leibrock C, Pandyra AA, Stournaras C, Wagner CA, Foller M (2018). Phosphate homeostasis, inflammation and the regulation of FGF-23. Kidney Blood Press Res..

[29] Bar L, Stournaras C, Lang F, Foller M (2019). Regulation of fibroblast growth factor 23 (FGF23) in health and disease. FEBS Lett.

