# Supplementary material for: C-terminal and intact FGF23 in kidney transplant recipients and their associations with overall graft survival
Source: BMC Nephrol. 2021 Apr 8;22:125. doi: 10.1186/s12882-021-02329-7 (PMC8033679; doi:10.1186/s12882-021-02329-7)
Supplement: Supplementary file 1 — Additional file 1. [file 12882_2021_2329_MOESM1_ESM.docx]

**C-terminal and intact FGF23 in kidney transplant recipients and their associations with overall graft survival**

Chang Chu, Saban Elitok, Shufei Zeng, Yingquan Xiong, Carl-Friedrich Hocher, Ahmed A. Hasan, Bernhard K. Krämer, Berthold Hocher

**Supp. Meta-analysis**

**S1. Method**

**S1.1 Data sources**

A systematic literature search was conducted to identify studies investigating the association between serum or plasm FGF23 and the composite outcome of all-cause mortality and graft loss in kidney transplant recipients in PubMed and Embase (last searches on 10 November 2020).

**S1.2 Study selection and eligibility criteria**

Two reviewers independently screened titles and abstracts and further assessed the full text of each potentially relevant study to determine eligibility for inclusion. Reviews, reports to congress, commentaries, case reports, editorials were excluded. Abstracts were reviewed and cohort studies in adults were selected for inclusion in the meta-analysis if they met the following criteria (1) adult KTRs cohort, (2) FGF23 was a key exposure of interest, (3) at least one clinical composite outcome was assessed, (4) and hazard risk (HR) and the corresponding 95% confidence interval (CI) or sufficient data to calculate them were provided. Studies were excluded if the sample size was less than 200 and the duration of follow-up was less than 12 months. Publications presenting preliminary results were excluded if the later one was available to avoid including duplicated data.

**S1.3 Data extraction and quality assessment**

Two independent authors extracted the following data from full-text articles: study size and population characteristics, FGF23 assay type (C-terminal or intact), total number of events, follow-up duration, and HRs or relevant outcomes.

The quality of included studies was assessed using the Newcastle-Ottawa scale. Studies that scored nine stars were rated to be at low risk of bias, a score of seven or eight stars was considered to be at medium risk and a score of six or less stars was rated as high risk of bias (Supplementary table 1).

**S1.4 Data analysis**

For each study, we aimed to extract from the primary publication, for each outcome, the HR yielded by the model that included the greatest number of covariates. Either fixed-effects model or, in the presence of heterogeneity, random-effects method was used in the pooled results. Heterogeneity across studies was assessed by testing with the I^2^-statistic, considering I^2^ <40%, 40-75% and>75% as an indication of low, moderate and high variability, respectively. Begg and the Egger test were performed to evaluate Potential publication bias in meta-analysis; however, the accuracy of these tests is low or may even be misleading when fewer than 10 studies. Data were expressed as HR and 95% CI. All analyses were performed using Stata/SE version 14.0 (StataCorp LP, College Station, Texas). Tests were two sided and a P value <0.05 was considered statistically significant.

**S2. Results**

**S2.1 Meta-analysis of the association between FGF23 and** **composite outcome in KTRs.**

The search strategy identified 409 potentially relevant studies from databases. After removal of duplicates, 370 citations were screened based on titles and abstracts. 356 Articles were excluded due to inappropriate population, exposure or outcome. 14 studies remained for full-text eligibility assessment. Five studies were excluded due to inappropriate article type. Four studies with < 200 participants were also excluded. One study reported association among FGF-23, ID and mortality (1), however, this could not be extracted as HRs. Finally, five studies (including our own study) were included in this meta-analysis, involving 3257 patients with 694 overall graft loss events (Supplementary table 2). The pooled HR (95% CI) of baseline circulating cFGF23 concentrations for composite outcome was 1.27 (95% CI, 1.01 to 1.61, p=0.041, I^2^=87.9%) (Supplementary figure 3). The Begg and Egger tests showed no evidence of publication bias among studies of cFGF23 and overall graft loss (Begg, p = 0. 071; Egger, p= 0.07).

**S3. Limitations**

This meta-analysis has several limitations. First, only 5 studies were included in this meta-analysis and synthesis of results were based on different adjustments from each study. Second, most FGF23 concentrations were tested only once instead of repeated measurement over time. Third, recruited transplant recipients are at variable durations after transplant. All of those could be potential explanations of the high heterogeneity.

**Supplementary table 1.** Newcastle-Ottawa scale of included studies.

| Author | QUALITY ASSESSMENT- NEWCASTLE OTTAWA QUALITY ASSESSMENT SCALE (COHORT STUDIES) | | | | | | | | |
| --- | --- | --- | --- | --- | --- | --- | --- | --- | --- |
|  | SELECTION | | | | COMPARABILITY | OUTCOME | | | TOTAL |
|  | Cohort representativeness (*) | Selection of non-exposed (*) | Ascertainment of exposure (*) | Incident event (*) | (**) | Assessment of outcome (*) | Length of follow-up (*) | Adequacy of follow-up (*) |  |
| Wolf M et al. | * | * | * | * | * | * | * | * | 8 |
| Baia LC et al. | * | * | * | * | * | * | * | * | 8 |
| Prakobsuk S et al. | * | * | * | * | - | * | * | * | 7 |
| Bienaimé F et al. | * | * | * | * | ** | * | * | * | 9 |
| Chu C et al. | * | * | * | * | * | * | * | * | 8 |

**Supplementary table 2.** Main characteristics of cohort studies in the meta-analysis.

| Author | Study Location | No. KTRs | Age (mean ±SD) | Gender Men (%) | Sample source | Median follow-up | Outcome | Events | FGF-23  Assay Type | Methods |
| --- | --- | --- | --- | --- | --- | --- | --- | --- | --- | --- |
| Wolf M et al. | Hungary | 984 | 51±13 | 561 (57%) | Serum | 37 months | Death and graft loss | D:87  GL:101 | cFGF23 | ELISA |
| Baia LC et al. | Netherlands | 593 | 52±12 | 320 (54%) | Plasma | 73.2 months | Death and graft loss | D:128  GL:54 | cFGF23 | ELISA |
| Prakobsuk S et al. | Thailand | 273 | 46±12 | 173 (63%) | Serum | 71.4 months | Death and graft loss | GL:41 | cFGF23 | ELISA |
| Bienaimé F et al. | France | 845 | 49±14 | 492 (59%) | Plasma | 71 months | Death and graft loss | D:105  GL:84 | cFGF23 | ELISA |
| Chu C et al. | Germany | 569 | 54±15 | 349 (61%) | Serum | 48 months | Death and graft loss | D:65  GL:37 | cFGF23 | ELISA |

KTRs, kidney transplant recipients, D, death; GL, graft loss.

**Supplementary table 3. TRIPOD Checklist: Prediction Model Development.**

| **Section/Topic** | **It Item** | **Checklist Item** | **Page** |
| --- | --- | --- | --- |
| **Title and abstract** | | | |
| Title | 1 | Identify the study as developing and/or validating a multivariable prediction model, the target population, and the outcome to be predicted. | 1 |
| Abstract | 2 | Provide a summary of objectives, study design, setting, participants, sample size, predictors, outcome, statistical analysis, results, and conclusions. | 2 |
| **Introduction** | | | |
| Background and objectives | 3a | Explain the medical context (including whether diagnostic or prognostic) and rationale for developing or validating the multivariable prediction model, including references to existing models. | 3-4 |
|  | 3b | Specify the objectives, including whether the study describes the development or validation of the model or both. | 3-4 |
| **Methods** | | | |
| Source of data | 4a | Describe the study design or source of data (e.g., randomized trial, cohort, or registry data), separately for the development and validation data sets, if applicable. | 4-5 |
|  | 4b | Specify the key study dates, including start of accrual; end of accrual; and, if applicable, end of follow-up. | 4 |
| Participants | 5a | Specify key elements of the study setting (e.g., primary care, secondary care, general population) including number and location of centres. | 4 |
|  | 5b | Describe eligibility criteria for participants. | 4-5 |
|  | 5c | Give details of treatments received, if relevant. | NA |
| Outcome | 6a | Clearly define the outcome that is predicted by the prediction model, including how and when assessed. | 5 |
|  | 6b | Report any actions to blind assessment of the outcome to be predicted. | 4-5 |
| Predictors | 7a | Clearly define all predictors used in developing or validating the multivariable prediction model, including how and when they were measured. | 5-7 |
|  | 7b | Report any actions to blind assessment of predictors for the outcome and other predictors. | 4-5 |
| Sample size | 8 | Explain how the study size was arrived at. | NA |
| Missing data | 9 | Describe how missing data were handled (e.g., complete-case analysis, single imputation, multiple imputation) with details of any imputation method. | NA |
| Statistical analysis methods | 10a | Describe how predictors were handled in the analyses. | 6-7 |
|  | 10b | Specify type of model, all model-building procedures (including any predictor selection), and method for internal validation. | 6-7 |
|  | 10d | Specify all measures used to assess model performance and, if relevant, to compare multiple models. | 6-7 |
| Risk groups | 11 | Provide details on how risk groups were created, if done. | NA |
| **Results** | | | |
| Participants | 13a | Describe the flow of participants through the study, including the number of participants with and without the outcome and, if applicable, a summary of the follow-up time. A diagram may be helpful. | 8 |
|  | 13b | Describe the characteristics of the participants (basic demographics, clinical features, available predictors), including the number of participants with missing data for predictors and outcome. | 7-8 |
| Model development | 14a | Specify the number of participants and outcome events in each analysis. | 8 |
|  | 14b | If done, report the unadjusted association between each candidate predictor and outcome. | 8-9 |
| Model specification | 15a | Present the full prediction model to allow predictions for individuals (i.e., all regression coefficients, and model intercept or baseline survival at a given time point). | 8-10 |
|  | 15b | Explain how to the use the prediction model. | 8-10 |
| Model performance | 16 | Report performance measures (with CIs) for the prediction model. | 8-10 |
| **Discussion** | | | |
| Limitations | 18 | Discuss any limitations of the study (such as nonrepresentative sample, few events per predictor, missing data). | 12-14 |
| Interpretation | 19b | Give an overall interpretation of the results, considering objectives, limitations, and results from similar studies, and other relevant evidence. | 11-14 |
| Implications | 20 | Discuss the potential clinical use of the model and implications for future research. | 13 |
| **Other information** | | | |
| Supplementary information | 21 | Provide information about the availability of supplementary resources, such as study protocol, Web calculator, and data sets. | See Supplementary file |
| Funding | 22 | Give the source of funding and the role of the funders for the present study. | 15 |

**Supplementary table 4.** Cox regression analysis for graft loss and all-cause mortality.

| Variable | Graft loss | | | | All-cause mortality | |
| --- | --- | --- | --- | --- | --- | --- |
|  | HR (95% CI) | P | | | HR (95% CI) | P |
| ***log C-terminal FGF23*** | | | | | | |
| Univariate analysis | 1.679 (1.448-1.946) | | | <0.001 | 1.257 (1.072-1.474) | 0.005 |
| Model 1 | 1.261 (1.021-1.556) | | | 0.031 | 1.121 (0.920-1.367) | 0.256 |
| Model 2 | 1.136 (0.918-1.406) | | | 0.240 | 1.183 (0.947-1.479) | 0.138 |
| Model 3 | 1.300 (0.880-1.920) | | | 0.188 | 1.266 (0.837-1.916) | 0.264 |
| Model 4 | 1.379 (0.906-2.098) | | | 0.134 | 1.258 (0.835-1.894) | 0.273 |
| Model 5 | 1.310(0 .870-1.972) | | | 0.197 | 1.234 (0.805-1.893) | 0.334 |
| Model 6 | 1.395 (0.893-2.177) | | | 0.143 | 1.227 (0.803-1.874) | 0.344 |
| ***log intact FGF23*** | | | | | | |
| Univariate analysis | 1.539 (1.324-1.790) | | <0.001 | | 1.082 (0.913-1.281) | 0.364 |
| Model 1 | 1.134 (0.940-1.368 | | 0.190 | | 0.919 (0.764-1.105) | 0.368 |
| Model 2 | 1.040 (0.857-1.263) | | 0.690 | | 0.949 (0.775-1.162) | 0.613 |
| Model 3 | 0.935 (0.655-1.336) | | 0.713 | | 1.051 (0.729-1.516) | 0.790 |
| Model 4 | 0.921 (0.621-1.364) | | 0.680 | | 1.011 (0.695-1.471) | 0.954 |
| Model 5 | 0.882 (0.609-1.277) | | 0.506 | | 1.022 (0.707-1.478) | 0.908 |
| Model 6 | 0.887 (0.598-1.314) | | 0.550 | | 0.991 (0.682-1.440) | 0.962 |

Models were adjusted for confounding factors. Model 1: eGFR; Model 2: eGFR, gender, age; Model 3: eGFR, gender, age, time post-transplantation, hemoglobin, albumin, donor’s age, cold ischemia time, log serum calcium, log serum phosphorus, log parathyroid hormone, urinary protein excretion; Model 4: model 3 + CRP; Model 5: model 3 + MCV + ferritin; Model 6: model 3 + CRP + MCV + ferritin. HR, hazard ratio; 95% CI, 95% confidence interval. eGFR, estimated glomerular filtration rate; CRP, C-reactive protein; MCV, mean corpuscular volume.


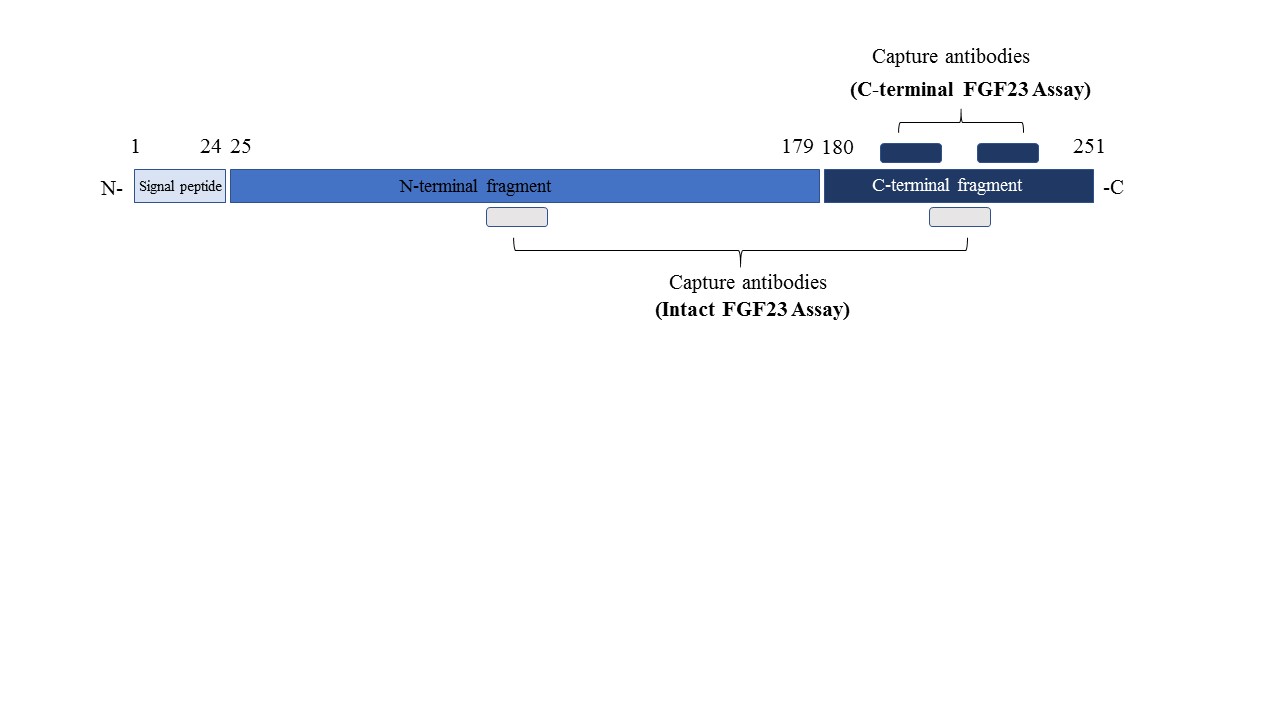


**Supplementary figure 1.** Full length FGF23 is a 251 amino acid peptide with a 24 amino acid signal peptide and 227 residues, is cleaved by subtilisin-like proprotein recognize 179Arg and 180Ser amino acid sequences within the FGF homology region present in the N-terminal part of the FGF-23 structure and produce a biologically inactive peptide which comprises an inactive N-terminal (amino acids 25-179) and C-terminal fragment (amino acids 180-251). Intact FGF23 assays use capture antibodies (light gray) that bind two epitopes that flank the proteolytic cleavage of FGF23 site, detect only biologically active FGF23 (amino acids 25-251). C-terminal assays use capture antibodies (dark blue) that bind two epitopes in the C-terminus of FGF23, detect not only biologically active FGF23, but also its inactive C-terminal fragments. The binding sites of the antibodies were not identical of intact FGF23/ cFGF23 assays from different companies and were only showed as approximate regions in the figure due to antibody epitope mapping data are not available.


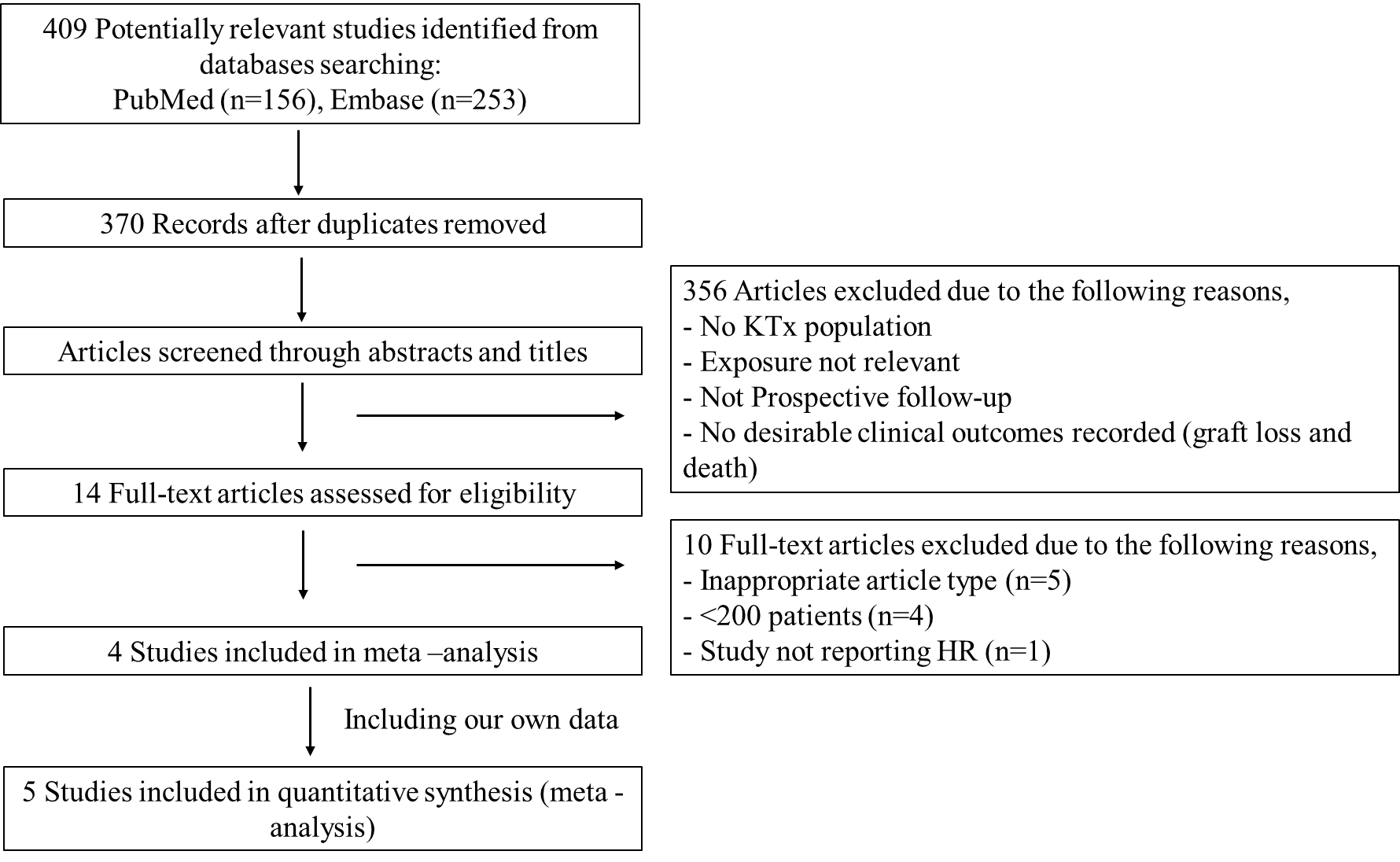


**Supplementary figure 2.** Flow diagram of studies through review.


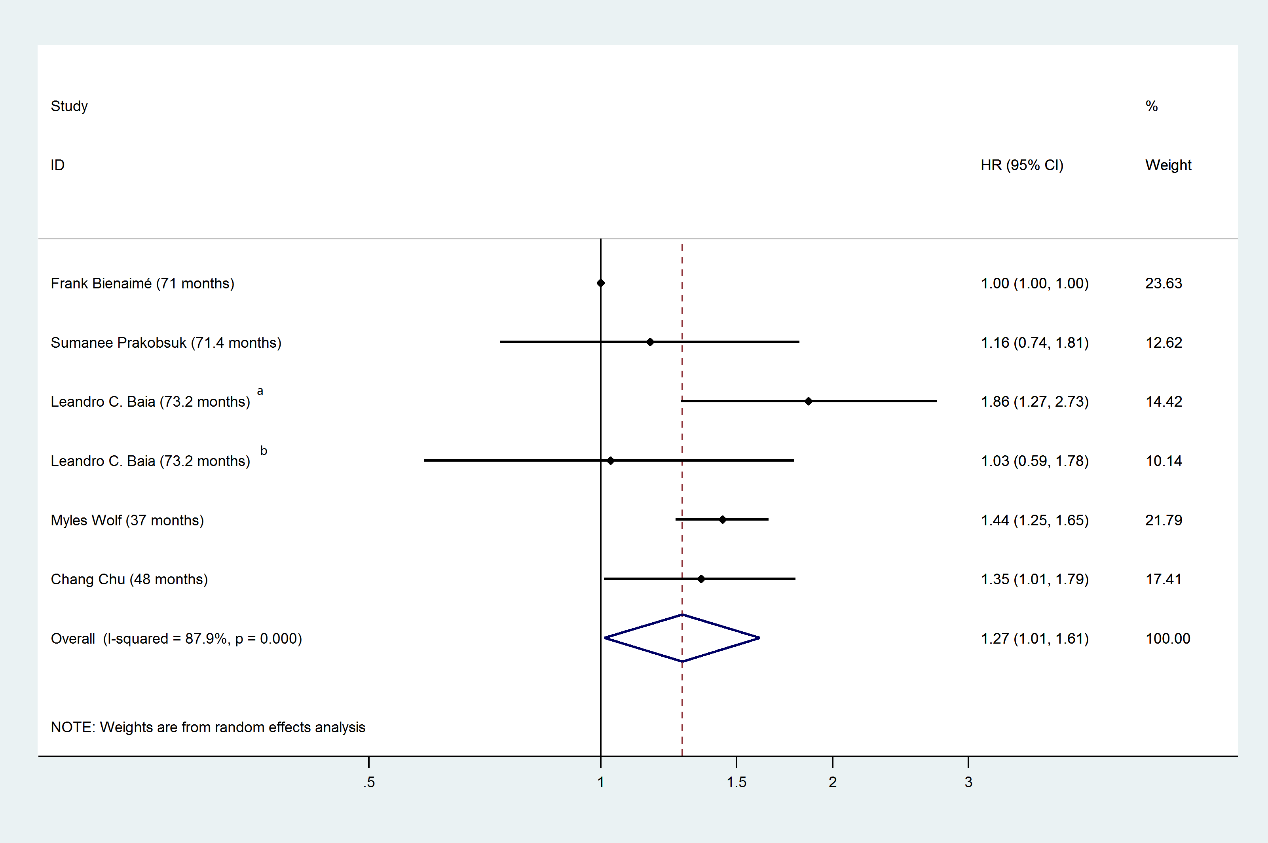


**Supplementary figure 3.** Forest plot for the association between cFGF23 and composite outcome of all-cause mortality and graft loss in kidney transplant recipients (HR, 1.27, 95% CI, 1.01 to 1.61, p=0.041; I^2^=87.9%). a, all-cause mortality; b, graft loss.

**References**

1. Eisenga MF, van Londen M, Leaf DE, Nolte IM, Navis G, Bakker SJL, et al. C-Terminal Fibroblast Growth Factor 23, Iron Deficiency, and Mortality in Renal Transplant Recipients. J Am Soc Nephrol. 2017;28(12):3639-46.
